# Sneezing-Induced Subclavian Arterial Rupture: A Case of Vascular Ehlers-Danlos Syndrome in a Child

**DOI:** 10.7759/cureus.51324

**Published:** 2023-12-30

**Authors:** Tomoya Hanada, Koji Kanno, Yusuke Ito, Yuji Yamagami, Kosuke Yoshizawa

**Affiliations:** 1 Department of Pediatric Emergency and Critical Care, Hyogo Prefectural Amagasaki General Medical Center, Amagasaki, JPN; 2 Department of Cardiovascular Surgery, Hyogo Prefectural Amagasaki General Medical Center, Amagasaki, JPN

**Keywords:** ehlers-danlos syndrome, critical care, col3a1, vascular eds, cardiovascular medicine, emergency medicine, arterial rupture, collagen disease, children, pediatrics

## Abstract

Vascular Ehlers-Danlos syndrome is a fatal disease caused by a type III collagen mutation that can result in the rupture of blood vessels, the intestinal tract, and/or the uterus. Despite being the most severe form of Ehlers-Danlos syndrome, it is not well known in the pediatric context because it rarely presents serious complications in childhood.

In this case, the patient experienced a subclavian artery rupture triggered by sneezing, which was initially managed with an endovascular stent. However, the descending aorta subsequently ruptured, and the patient died. Traditionally, surgical or endovascular treatments have been avoided due to the inherent fragility of blood vessels. Nevertheless, favorable outcomes have been documented with a wait-and-see surgical approach or endovascular treatment, especially when the diagnosis precedes the onset of vascular complications. Notably, celiprolol, a partial β2-agonist and β1-blocker, has demonstrated efficacy in preventing vascular complications. Therefore, early diagnosis plays a pivotal role. Raising awareness about this syndrome, along with its management and prophylaxis, holds the potential to enhance the survival rate.

## Introduction

Vascular Ehlers-Danlos syndrome (vEDS) stands as a life-threatening condition stemming from mutations in the type III collagen gene (COL3A1). This genetic anomaly precipitates the potential rupture of blood vessels, intestines, and the uterus [1,2]. The associated mortality rate, particularly linked to vascular complications, ranges significantly from 20% to 40%, with a staggering 68% for aortic lesions [3]. Despite being the most severe variant of Ehlers-Danlos syndrome, its prevalence in the pediatric domain is limited, as serious complications seldom manifest during childhood. Celiprolol, a partial β2-agonist and β1-blocker, has demonstrated efficacy in preventing vascular complications [4,5]. Consequently, early diagnosis and rigorous blood pressure management before complications emerge are strongly advocated. While characteristic clinical manifestations are often subtle in childhood [6], vigilance should not waver regarding indicators such as easy bleeding or hypermobility of small joints. Additionally, maintaining an awareness of this condition in routine medical practice is imperative to facilitate timely and accurate differential diagnoses.

## Case presentation

Herein, we present a case of a 12-year-old male patient with a body mass index of 21.4 kg/m^2^, who experienced sudden and intense pain near the right scapula following a sneeze. His medical history revealed a tendency for bruising, but no familial history of similar complaints. Upon experiencing pain in the right scapula after sneezing, the patient sought immediate medical attention due to difficulty moving.

**Figure 1:**
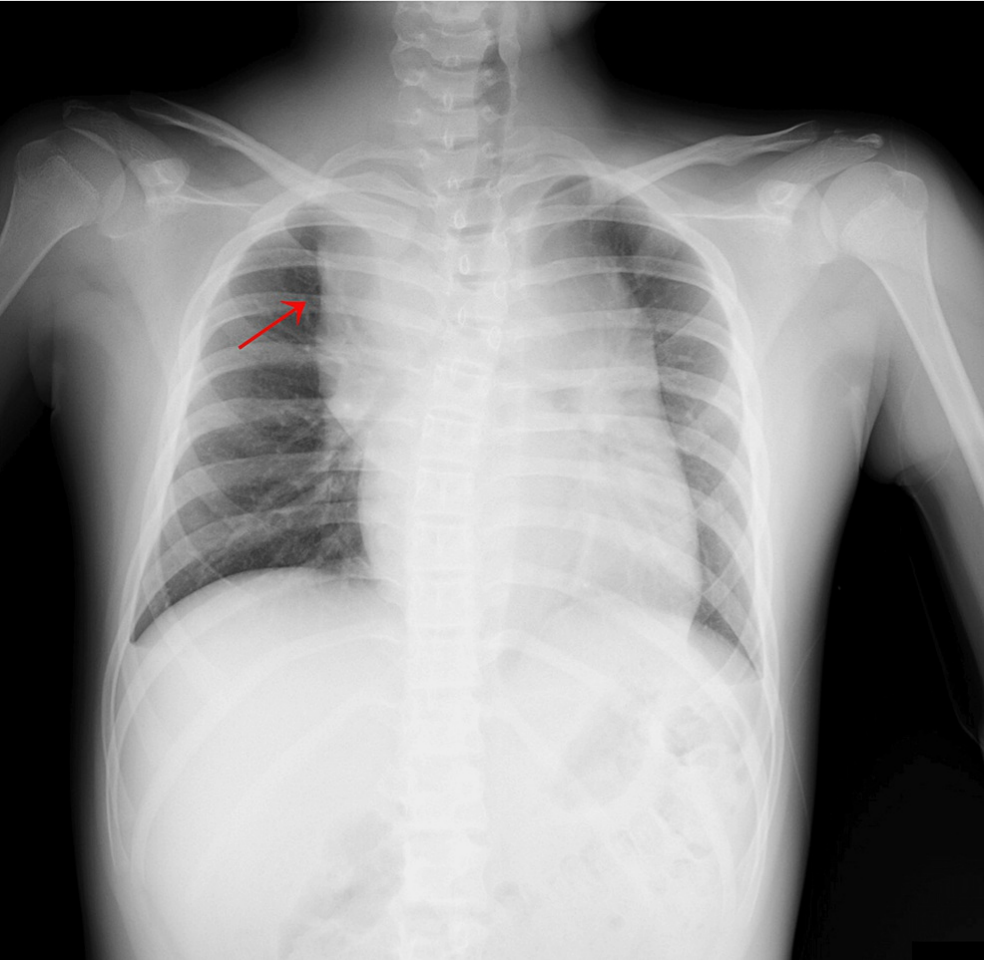
Chest X-ray on arrival Chest X-ray showing an enlarged superior mediastinum (red arrow).

**Figure 2:**
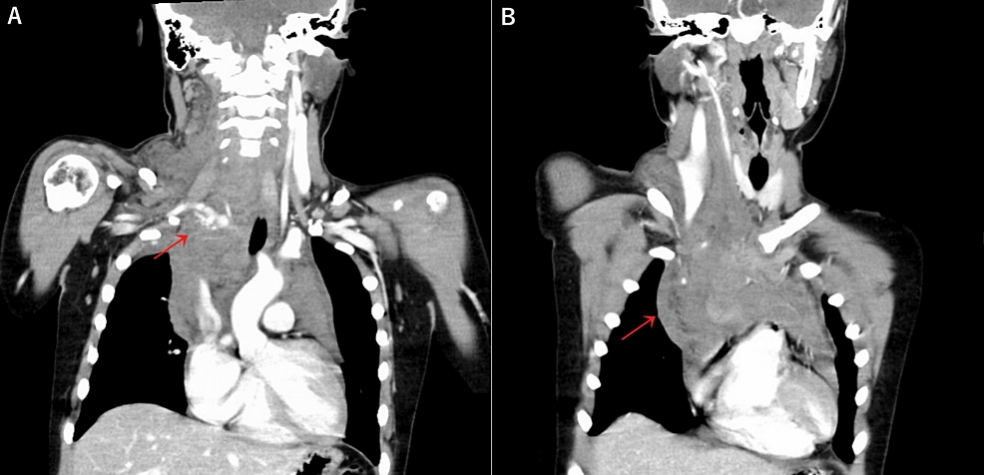
Chest contrast-enhanced CT on arrival (A) Active bleeding from the proximal portion of the right subclavian artery. (B) Hematoma in the mediastinum.

Upon arrival at our emergency department, his respiratory rate was 27 breaths per minute, his oxygen saturation was 100% on room air, his heart rate was 120 beats per minute, and his blood pressure was 121/82 mmHg and 106/56 mmHg in his left and right upper arms, respectively. His temperature was 36.8°C, and his Glasgow Coma Scale score was eye response 4, verbal response 4, and motor response 6. The patient exhibited a mass in the right supraclavicular fossa, accompanied by pain radiating from the right neck to the scapula. Notably, hypermobility of the wrist and finger joints was observed. The observed difference in blood pressure between the right and left upper extremities raised suspicion of arterial hemorrhage. Chest radiography revealed an enlarged superior mediastinum (Figure 1), while contrast-enhanced computed tomography (CT) revealed active bleeding from the proximal portion of the right subclavian artery, forming a hematoma in the mediastinum (Figure 2).

**Figure 3:**
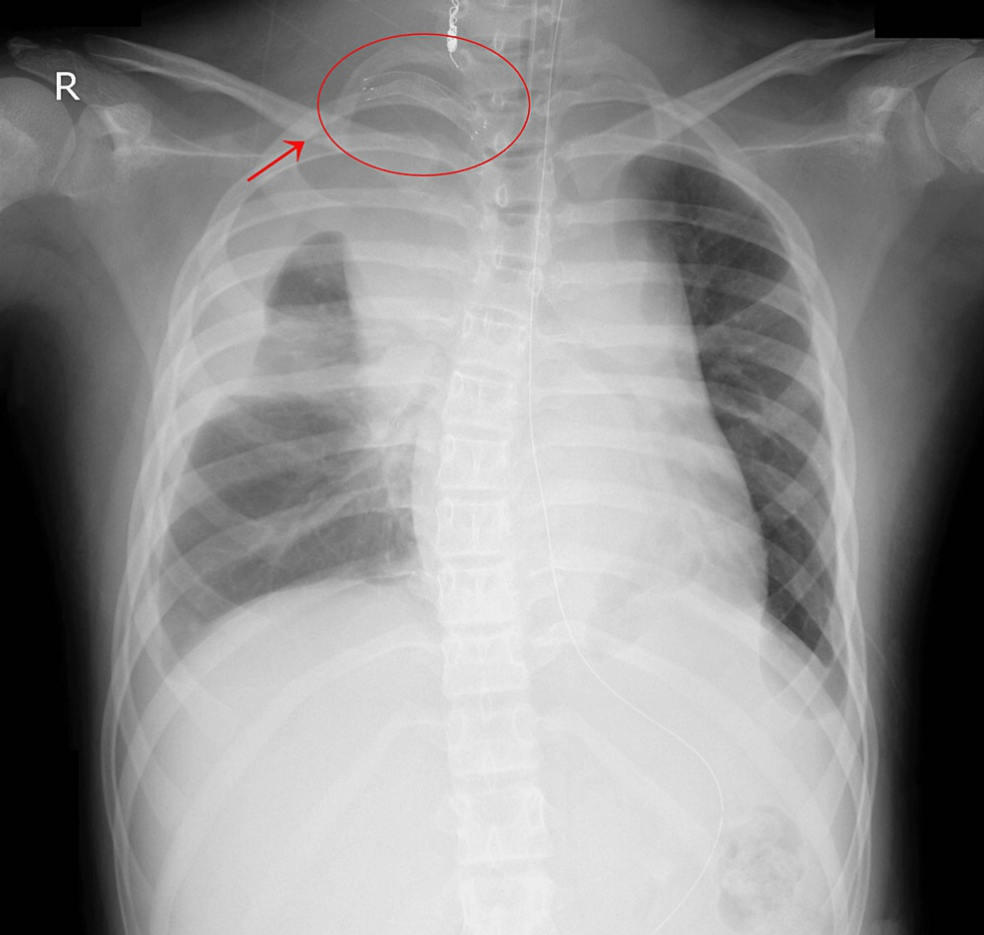
Chest X-ray after stenting on day 0 Chest X-ray showing the stent placed in the right subclavian artery (red arrow and red circle) on day 0.

The patient rapidly developed hemorrhagic shock, prompting the urgent placement of a stent in the right subclavian artery to halt the bleeding. Subsequently, the patient was admitted to the pediatric intensive care unit (Figure 3).

**Figure 4:**
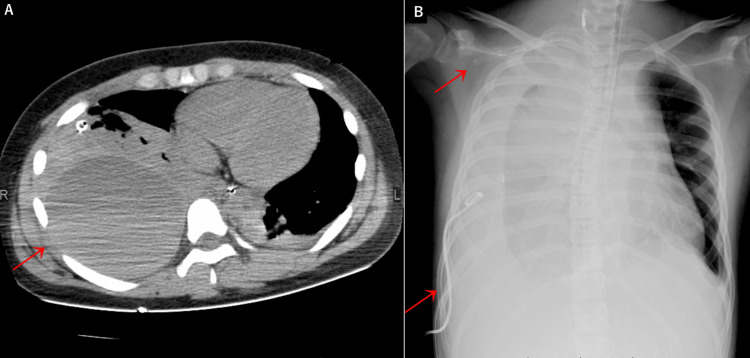
Representative images of a right hemothorax and a large right extrapleural hematoma (A) Chest CT scan showing a right hemothorax and a large right extrapleural hematoma (red arrow). (B) Chest X-ray showing additional stenting of the right subclavian artery and drainage of the hemothorax and extrapleural hematoma (red arrow).

However, by day two, there was a recurrence of hypotension, leading to shock. Contrast-enhanced CT imaging revealed a right hemothorax and a sizable, right extra-pleural hematoma. In response, additional stenting of the right subclavian artery and drainage procedures for the hemothorax and extra-pleural hematoma were performed (Figure 4).

**Figure 5:**
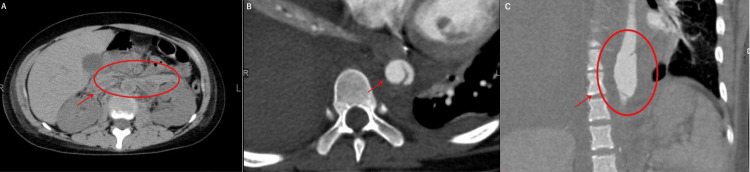
Representative images of rupture of the descending aorta (A) CT scan showing hematoma around the ruptured descending aorta (red arrow and red circle). (B) Axial view of contrast-enhanced CT scan showing dissection cavity (red arrow). (C) Coronal view of contrast-enhanced CT scan showing thoracic aortic aneurysm (red arrow and red circle).

Following these interventions, the patient's blood pressure stabilized, but persistent poor oxygenation with a P/F ratio of 50-60 was noted due to compression of the right lung and mediastinum. Extubation occurred on day 10, after which the patient received management through non-invasive positive-pressure ventilation, transitioning to high-flow nasal cannulas on day 15. The patient commenced oral intake on day 16. However, on day 17, the patient reported sudden abdominal pain and subsequent imaging revealed an aortic dissection extending from the descending thoracic aorta to the abdominal aorta and a ruptured thoracic aortic aneurysm (Figure 5). Tragically, the patient succumbed to these complications on the same day.

At the time of arrival, hereditary connective tissue diseases, such as vEDS, Marfan syndrome, and Loeys-Dietz syndrome, or tumor-related hemorrhage, were mentioned in our differential. The occurrence of a ruptured artery at a young age further heightened suspicion of vEDS. Following admission, genetic testing was promptly initiated, and approximately six weeks later, a pathological genetic mutation in the COL3A1 gene was identified, conclusively confirming the diagnosis.

## Discussion

vEDS is inherited as an autosomal dominant trait and is a rare disease with a frequency of 1/50,000 to 1/250,000 individuals. It is the most severe form of Ehlers-Danlos syndrome, causing rupture of the intestinal tract, uterus, blood vessels, and other organs. Approximately 25% of patients develop serious complications such as ruptured arteries by the age of 20 years, whereas over 80% experience it by 40 years. It is a fatal disease, with a median age of death of 48 years [2]. However, compared with those associated with other types of Ehlers-Danlos syndrome, the skin and joint symptoms are mild [6].

Herein, we present the case of a pediatric patient with vEDS who, despite multidisciplinary treatment efforts, succumbed to the condition. This case was marked by the swift development of arterial lesions, prompting a discourse on treatment approaches, strategies, and preventive measures for vascular lesions.

In this particular case, hypotensive events occurred on days zero and two. Bleeding from the ruptured subclavian artery into the mediastinum and thoracic cavity likely precipitated the hypotensive event on day 0. On day two, presumed causes included hemorrhagic and obstructive shock due to severe mediastinal compression from rebleeding in the subclavian artery and bleeding from the intercostal arteries. In the management of vEDS, conservative treatment is advocated for arterial collapse [7]. However, interventional radiology (IVR) or surgical treatment may be warranted in specific cases to preserve the life of the patient. In this instance, we opted for IVR, anticipating that the patient could be saved if the event affected only the subclavian artery. Tragically, the patient succumbed on day 17 due to a rupture of the descending aorta, distant from the subclavian artery. In vEDS, a fatal vascular event termed a remote vascular catastrophe may occur at a site distant from the original lesion following endovascular treatment or surgery [8]. This was deemed the scenario in this case. Although the pathogenesis of this remote vascular catastrophe remains unclear, the patient underwent stenting via the right femoral artery. Moreover, the vascular burden caused by arteriography and IVR may be a contributing factor.

The primary approach to preventing vascular events involves early disease detection and rigorous blood pressure control in specialized cardiovascular facilities. However, options such as wait-and-see surgical vascular repair and endovascular treatment exist [9,10]. In this case, the patient exhibited a history of shoulder joint dislocation, a propensity to bruise, and physical indications of small joint hypermobility, suggesting a potential connective tissue disease. While proposing genetic testing for connective tissue diseases in general pediatric practice is challenging without vascular, intestinal, or uterine complications, certain regions are witnessing a rise in hospitals specializing in outpatient clinics for such diseases, encouraging active referral and screening.

The patient's blood pressure was generally controlled at a systolic pressure of 130 mmHg or less by continuous intravenous infusion of nicardipine and oral administration of celiprolol, losartan, and amlodipine. Because of concerns about rebleeding, the patient was immobilized for more than one week longer to confirm that the arterial bleeding had not progressed, after which he was extubated. Celiprolol, a partial β2-agonist and β1-blocker, has been shown to enhance collagen production, maintain collagen fiber continuity in arterial walls, and decrease mechanical stress associated with pulsation [4]. The Beta-Blockers in Ehlers-Danlos Syndrome Treatment (BBEST) trial and other studies have reported benefits and safety in treating this disease [4,5,11]. In this case, the drug was administered from day 12; however, given the necessary duration for collagen production (drug titration every three to six months with a median follow-up of 22 months in the study by Baderkhan et al. [5]), it should be administered for an extended period before the onset of vascular events.

Regarding the reason that similar episodes did not occur until the age of 12 years, mechanical stress from pulsation may gradually accumulate concurrently with congenital vascular fragility, increasing in frequency with advancing age, as described above. In this regard, vascular events may be less likely to develop stochastically during childhood.

Historically, surgical or endovascular treatment was avoided due to vessel fragility. Yet, favorable outcomes have been reported with wait-and-see surgical repair or endovascular treatment when the diagnosis precedes vascular complications [9,10]. Early diagnosis is particularly crucial as the survival rate for patients undergoing emergency surgery is minimal [3].

## Conclusions

We encountered a pediatric case of vEDS with arterial collapse. Favorable outcomes have been documented with a wait-and-see surgical approach or endovascular treatment, especially when the diagnosis precedes the onset of vascular complications. Notably, celiprolol has demonstrated efficacy in preventing vascular complications. Therefore, early diagnosis plays a pivotal role. Raising awareness about this syndrome, along with its management and prophylaxis, holds the potential to enhance the survival rate.
